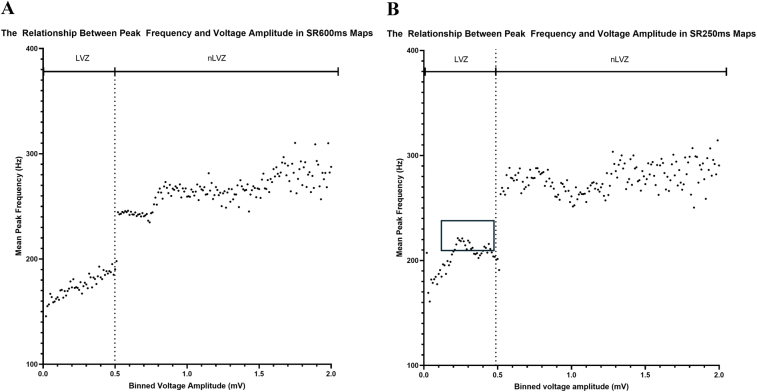# Peak frequency can be effectively used to characterize scar in atrial fibrillation

**DOI:** 10.1016/j.hroo.2024.12.011

**Published:** 2025-01-09

**Authors:** Sayed Al-Aidarous, Caterina Vidal Horrach, Caroline Roney, Charles Butcher, Ross J. Hunter, Shohreh Honarbakhsh

**Affiliations:** 1 Electrophysiology Department, Barts Heart Centre, Barts Health NHS Trust, London, United Kingdom; 2 UCL Institute of Cardiovascular Science, London, United Kingdom; 3 Queen Mary University of London, London, United Kingdom

**Keywords:** Atrial fibrillation, Substrate, Voltage assessment, Novel parameters, Peak frequency

## Abstract

**Background:**

Characterizing atrial fibrillation (AF) substrate can guide ablation strategies.

**Objective:**

A novel parameter, peak frequency (PF), was evaluated in its ability to characterize the substrate in AF.

**Methods:**

Patients undergoing persistent AF ablation were included. Patients had omnipolar voltage (OV) and PF maps in AF and bipolar voltage (BV) maps in sinus rhythm (SR) at pacing intervals of 600 and 250 ms. PF was evaluated at sites of fixed remodeling (low voltage zones [LVZs] across all maps), functional remodeling (LVZs in AF OV and SR BV 250 ms maps) and non-LVZs. *PF* was defined as the highest frequency detected in the electrogram.

**Results:**

In 40 patients, the average voltage in AF OV maps differed significantly from that in SR BV 600 ms maps (0.49±0.76 mV in AF OV vs 1.12±0.97 mV SR BV 600 ms; *P*<.001) but not SR BV 250 ms maps (0.49±0.76 mV in AF OV vs 0.52±0.84 mV SR BV 250 ms; *P*=.10). PFs of ≥244 and ≤214 Hz were predictive of non-LVZs (odds ratio [OR] 3.91; *P*<.001) with an area under the curve (AUC) of 0.71 and of fixed remodeling (OR 17.67; *P*<.001) with an AUC of 0.90, respectively. A PF between 215 and 236 Hz was predictive of functional remodeling (OR 2.83; 95% confidence interval 2.71–2.95; *P*<.001) with an AUC of 0.76. A majority of LVZs identified only in AF OV maps exhibited PF compatible with that seen in non-LVZs, suggesting that PF analysis can pinpoint potential overestimations of LVZs.

**Conclusion:**

PF can effectively discern between sites of fixed remodeling, functional remodeling, and potential overestimations of LVZs. PF may thereby aid in better characterization of the substrate in AF.

**Key Findings**

▪ This is the first study that has extensively characterized the substrate in atrial fibrillation (AF) in the form of fixed and functional remodeling.
▪ This study has shown that omnipolar voltage (OV) maps in AF are significantly different from sinus rhythm (SR) bipolar voltage (BV) maps at a pacing interval (PI) of 600 ms. However, these differences were not significant when comparing with SR at a PI of 250 ms, suggesting that AF OV maps correlate better with SR maps obtained at higher atrial rates and thereby considering both fixed and functional remodeling.
▪ A novel parameter, peak frequency (PF), was used to characterize the substrate in AF. PF mapping effectively differentiated between non–low voltage zones (nLVZs), fixed remodeling sites, and functional remodeling sites. PFs of ≤214 and ≥244 Hz were highly predictive of fixed remodeling and nLVZs, respectively. A PF between 215 and 236 Hz was highly predictive of functional remodeling. Thereby, the combination of OV and PF measurements in AF will more accurately allow the characterization of the substrate in AF.
▪ AF OV maps identified additional low voltage zones (LVZs) that were not seen on SR BV 600 ms and SR BV 250 ms maps, which is possible because of the underestimation of voltage due to mapping in AF. This is most likely the case as PF at these sites was not significantly different from PF obtained at nLVZs in AF OV maps. Thereby, PF can also be used to identify sites with potentially overestimated LVZs in AF.

## Introduction

Atrial fibrillation (AF) poses a significant health care challenge because of its rapidly rising prevalence^1^ and associated increase in morbidity and mortality.^2^ Despite notable advances in mapping techniques and a wealth of research, the efficacy of AF ablation procedures remains modest at best and leaves room for substantial improvement.^3^ While electrical isolation of the pulmonary veins (PVs) constitutes the fundamental strategy in AF ablation,^4^ there is increasing focus on targeting additional propagators of AF. Initial experience in the identification and targeted ablation of the proarrhythmic substrate, known as substrate modification, has yielded promising results.^5^

Low voltage zones (LVZs) correlate with regions of atrial fibrosis and scar^6^ and are key contributors to proarrhythmic substrate and independently predict AF recurrence.^7^ Targeting these sites with ablation has improved freedom from arrhythmia.^8^ Identification of these sites typically relies on bipolar peak-to-peak voltages, which requires a perpendicular wavefront of activation to the dipole.^9^^,^^10^ However, because of the disorganized nature of atrial excitation within AF, this directionality is inconsistent and may lead to overestimation of LVZs.^11^ Omnipolar voltage (OV) mapping can correct for wavefront directionality and electrogram (EGM) fractionation^12^ and correlates more accurately with voltage maps seen in sinus rhythm (SR).^13^ Additional methodologies have been used to better stratify scar in accordance with mechanistic importance in AF.^14^^,^^15^ Peak frequency (PF) mapping is a novel methodology distinct from dominant frequency (DF) analysis that annotates the highest frequency associated with intracardiac EGMs. Whether PF in conjunction with OV mapping can better characterize the substrate remains unclear.

Mapping during AF with OV unveils additional LVZs not seen on bipolar voltage (BV) maps acquired in SR.^13^ These additional LVZs may denote sites of rate-dependent proarrhythmic substrate or “functional remodeling” as opposed to scar. This highlights the composition of LVZs detected in OV maps during AF, which may be a combination of fixed and functional remodeling sites. Until now, substrate modification studies have primarily concentrated on homogenization of scar identified at baseline sinus rates,^16^ leaving the mechanistic significance of functional remodeling sites largely unexplored. Therefore, the ability to differentiate between sites of fixed and functional remodeling, without the need for additional voltage mapping at higher rates in SR, is of merit. Further understanding of the atrial remodeling process in AF can provide insights into AF mechanisms and thereby guide ablation strategies.

The study aimed to determine whether a novel parameter, PF, obtained in AF can be used to better characterize the substrate in AF and allow LVZs in AF to be characterized into fixed and functional remodeling. The study also aimed to determine whether PF can distinguish sites of low voltage, which is secondary to underestimation of voltage because of mapping in AF.

## Methods

Patients undergoing catheter ablation for persistent AF (<24 months and no previous AF ablation) were prospectively included. Exclusion criteria included age < 18 years or reversible cause of AF. Patients provided informed consent for their study involvement, which was approved by the UK National Research Ethics Service (22/PR/0961). The study was prospectively registered on ClinicalTrials.gov (NCT05633303). Procedures were performed under either conscious sedation or general anesthesia as per the clinician’s and patient’s preference. All procedures were performed on uninterrupted anticoagulation therapy with heparin administration during the procedure to maintain an activated clotting time of >300 seconds. All patients had β-blockers and antiarrhythmic drugs discontinued 5 days before the procedure. Because of the long half-life of amiodarone, patients on amiodarone had this continued.

### Electrophysiological mapping

#### Scar assessment

EnSite X (Abbott, Chicago, IL) was used as the 3-dimensional mapping system. Left atrial (LA) anatomic maps were created using the HD Grid mapping catheter (Abbott). All patients had high-density OV and PF maps created in AF using the HD Grid mapping catheter (Online Supplemental Methods). A proprietary algorithm (EnSite OT Near Field, Abbott) was used for OV assessment. For OV assessment, signals were obtained from 3 noncolinear electrodes that make up a clique. These are used to calculate the BV in all directions over 360°. The BV with the largest bipolar peak-to-peak voltage is then used to compute a local virtual bipolar signal that represents the OV. Following this, all peak-to-peak voltage points identified within a 1-mm sphere were identified. The voltage point with the highest OT certainty (numerical value ranging from 0 to 1, indicating how certain the calculated activation direction is) and largest voltage amplitude is then used for the final OV.^13^

Points that were ≥5 mm from the geometry surface were filtered as not being in contact with the myocardium, and points acquired were respiratory gated to optimize the accuracy of anatomical localization. A minimum of 5000 OV points were collected per patient with the aim to ensure adequate atrial coverage. The interpolation threshold was set to 5 mm for surface color projection, and points were collected aiming for complete LA coverage (ie, with no area >5 mm from a data point). *LVZs* were defined as sites with a voltage <0.5 mV.^17^ A decapolar catheter (Boston Scientific, Malaborough, MA) was positioned in the coronary sinus (CS). The TactiFlex ablation catheter (Abbott) was used for ablation. Following the voltage map in AF and before ablation, patients underwent direct current cardioversion to SR. All patients had a repeat BV map created in SR with atrial pacing at pacing intervals (PIs) of 600 and 250 ms to consider the atrial rates in AF. In this study, atrial pacing at a PI of 600 ms was used rather than a nonpaced sinus map to assess for fixed remodeling. This was to ensure that pacing at a fixed heart rate was performed to allow consistency across patients. Atrial pacing at a PI of 250 ms was used to identify functional remodeling. This pacing rate was used to mimic similar heart rates that are seen in AF while also minimizing the risk of AF recurrence during pacing. These BV maps were created with pacing from 2 sites—left atrial appendage (LAA) and distal CS—to ensure that the pacing site did not influence voltage assessment. All patients had BV maps created with LAA and CS pacing. The pacing site was consistent between the 600 and 250 ms maps to ensure that the impact on voltage was not secondary to fiber orientation and wavefront activation. The same criteria used for OV maps were used for BV maps. The BV map in SR created with atrial pacing at a PI of 600 ms (BV 600 ms) was used to identify sites of fixed remodeling, that is, LVZs. The BV map in SR created with atrial pacing at a PI of 250 ms was used to identify sites of functional remodeling.

**Figure 1:**
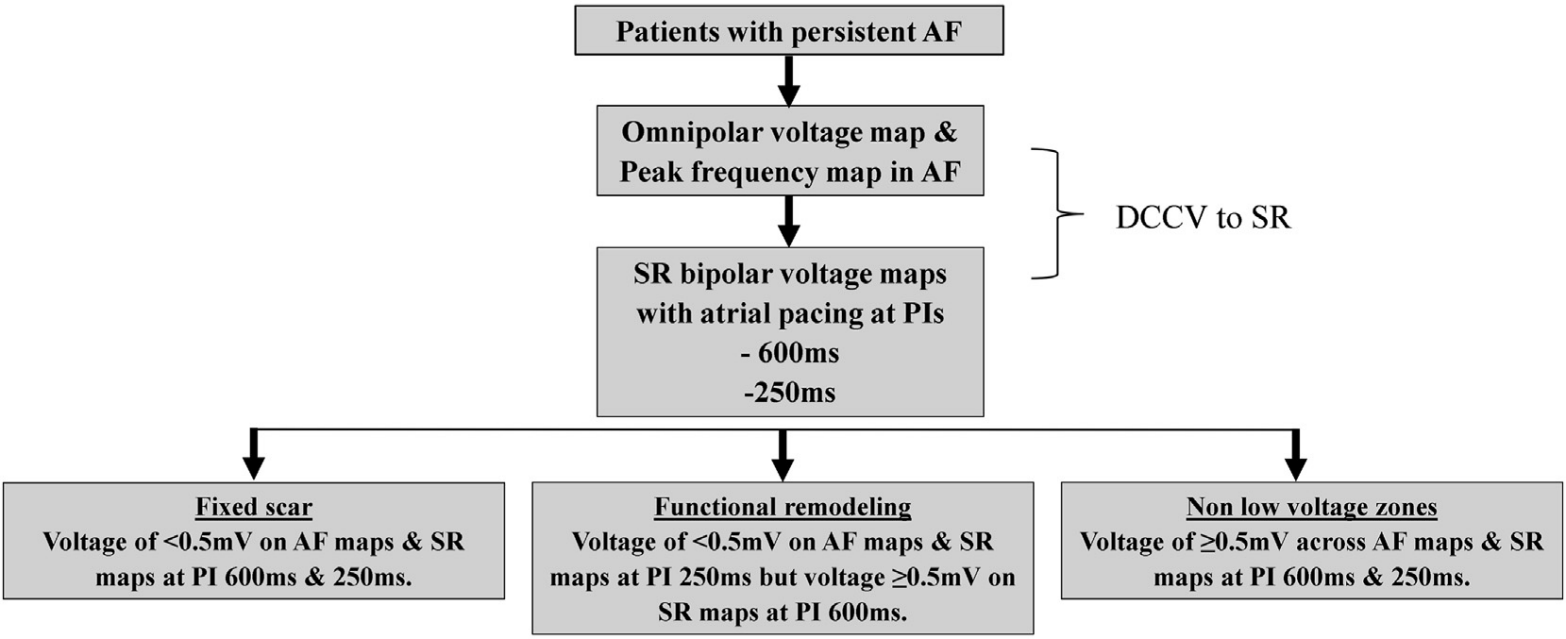
Study methodology diagram. AF = atrial fibrillation; DCCV = direct current cardioversion; PI = pacing interval; SR = sinus rhythm.

The *LA body* was defined as the LA excluding mitral valve annulus and PVs. The average voltage and proportion of LA body area occupied by LVZs were compared on AF OV maps and SR BV maps with atrial pacing at PIs of 600 ms (SR BV 600 ms maps) and 250 ms (SR BV 250 ms maps) to allow the assessment of fixed and functional remodeling. *Fixed remodeling* was defined as sites that demonstrated a voltage of <0.5 mV on SR BV 600 ms maps, AF OV maps, and SR BV 250 ms maps. *Sites of functional remodeling* were defined as sites that demonstrated a voltage of <0.5 mV on AF OV maps and SR BV 250 ms maps but a voltage of ≥0.5 mV on SR BV 600 ms maps (Figure 1). *Non-LVZs* (nLVZs) were defined as sites that demonstrated a voltage of ≥0.5 mV on AF OV maps, SR BV 600 ms maps, and SR BV 250 ms maps. Sites demonstrating a voltage of <0.5 mV on AF OV maps but not on SR BV 600 ms and SR BV 250 ms maps were also identified and termed as *AF-only LVZs*.

To review the differences in voltage at an anatomical site on the 3 voltage maps, voltage points were coregistered in accordance with xyz coordinates using an automated script in MatLab (MathWorks, Natick, MA). A *point* was defined as colocating to another point if they were within a geodesic distance of <3 mm. The anatomical distribution of voltage discrepancies was also determined using a 6-segment model of the atria (anterior, lateral, septal, posterior, inferior, and roof).

#### PF assessment

**Figure 2:**
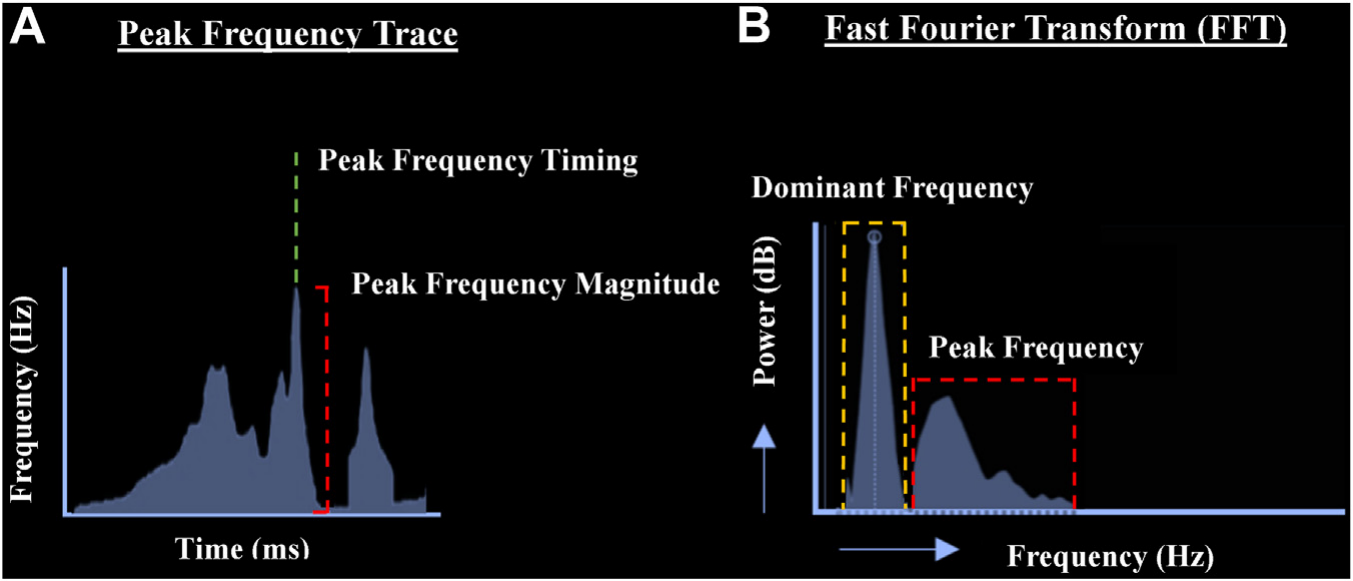
Methodology used for peak frequency (PF) analysis and the difference to the dominant frequency (DF). **A:** PF trace that reflects a measure of the highest signal frequencies detected in the intracardiac electrograms (EGMs) as a function of time. From the PF trace, 2 measures are obtained and assigned to each EGM point: (1) PF timing (*green line*), which is derived from the time of the highest detected frequency peak. (2) PF magnitude (*red line*), which represents the highest frequency detected in the EGM. **B:** Difference between PF and DF. It shows an EGM and its corresponding fast Fourier transform (FFT). The DF range (*dashed orange box*) represents the frequency of highest energy (typically low frequency), whereas the PF range (*dashed red box*) represents the highest frequencies in the EGM, often of low energy.

Using the OT near-field algorithm within the EnSite X mapping system (Abbott), the highest PF associated with mapped intracardiac EGMs were automatically measured and annotated.^18^ As the complex EGM consists of a summation of both near-field and far-field signal components, PF represents a novel measure of morphological component sharpness in the EGM serving to discriminate near-field from far-field components. Spanning the time course of each mapped EGM, a PF trace serves to track the highest EGM frequencies as a function of time (Figures 2A and 2B). PF is a wavelet-derived frequency in which the signal is decomposed into brief oscillations, based on scaled and time-shifted functions of a time-localized mother wavelet. From the PF trace, 2 measures are obtained and assigned to each EGM point: (1) the highest PF magnitude measured in hertz and (2) the local activation time corresponding to the instance of the highest PF magnitude (Figures 2A and 2B).

PF uses wavelet transform, an advanced mathematical signal processing technique. By breaking down intracardiac EGMs into their constituent frequencies while preserving temporal localization, wavelet analysis offers a unique dual perspective: it reveals not only which frequencies are present but also precisely when they occur. This combined time-frequency resolution distinguishes it from fast Fourier transform, which lacks temporal specificity and is therefore unable to determine the exact timing of different frequency components within the signal.

**Supplemental Figure 1:**
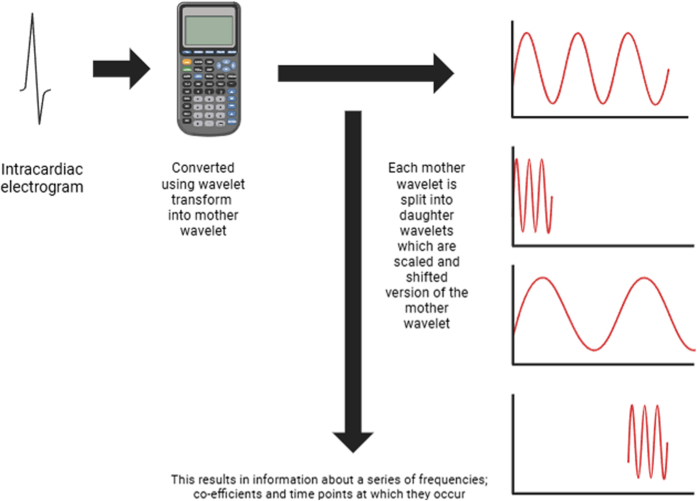


Wavelet analysis begins with the selection of a mother wavelet, a waveform specifically chosen to match the characteristics of cardiac signals. The EGM is then processed through a series of filters, transforming it into a collection of daughter wavelets. These daughter wavelets represent different frequency bands and are scaled and shifted versions of the original mother wavelet, allowing them to capture different frequencies while maintaining temporal information (Online Supplemental Figure 1). This feature is particularly valuable in capturing transient high-frequency components. The transformation produces a hierarchical set of wavelet coefficients, which quantify how much each daughter wavelet contributes to the overall signal. The resulting representation is composed of these coefficients, providing a detailed map of the energy present in the EGM across various frequencies, as well as when each frequency occurs. This dual representation of frequency content and its temporal distribution makes wavelet analysis and PF particularly useful. In addition, the original EGM can be reconstructed entirely from the wavelets and their coefficient, indicating that the underlying data remain intact and are not fundamentally altered. Rather, wavelet analysis repackages the data providing potentially greater insight into the inherent characteristics of the original signal.

In contrast to the wavelet-derived PF, the traditional method of frequency analysis using fast Fourier transform decomposes the EGM signal into infinite length sine and cosine waves of variable frequency and focuses on DFs, that is, those that contribute most significantly to the overall energy of the signal. However, as sine and cosine waves extend indefinitely, they do not allow the temporal localization of signals. Further to this, whereas DF reflects the highest energy often of lower frequency, PF reflects the highest frequency often of lower energy (Figures 2A and 2B). By using wavelet analysis, PF mapping can focus on the highest frequencies present within the EGM, even if they contribute less energy overall. This focus on high-frequency components, combined with precise timing information, is particularly advantageous for distinguishing between near-field and far-field signals within the EGM. Such differentiation is crucial in identifying subtle electrical activity that might point to areas vulnerable to arrhythmia initiation or maintenance.

#### Relationship between PF and voltage

PF values obtained in AF were coregistered to voltage points on AF OV, SR BV 600 ms, and SR BV 250 ms maps. PFs at nLVZs, LVZs defined as fixed remodeling, and LVZs defined as functional remodeling were determined. Differences in PF at these sites were evaluated.

### Ablation approach

All patients underwent PV isolation (PVI) with bilateral wide area circumferential ablation using radiofrequency ablation. PVI was achieved with lesions placed 5–10 mm outside the venoatrial junction, aiming for isolation as ipsilateral PV pairs. The anterior border of the left PVs was ablated on the LAA ridge where possible or on the appendage side of the ridge for cases where this was unstable. Lesions were delivered on the venous side of the appendage ridge only where this was necessary to isolate PVs. Ablation was performed with 45 W, 15 seconds anteriorly and 12 seconds posteriorly. Further ablation was performed if AF organized into an atrial tachycardia (AT) or there was previous documentation of AT.

### Statistical analysis

All statistical analyses were performed using SPSS version 25 (IBM Corporation, Armonk, NY). Continuous variables are displayed as mean ± SD or median (interquartile range). Categorical variables are presented as number (percentage). The Fisher exact test was used for the comparison of nominal variables. The Student *t* test or its nonparametric equivalent Mann-Whitney test was used for the comparison of continuous variables. One-way analysis of variance was performed to compare parameters during multigroup comparison. Binary logistic regression was used to determine the ability of PF to predict nLVZs, fixed remodeling, and functional remodeling. Receiver operating characteristic analysis was performed to determine the diagnostic accuracy of PF in predicting nLVZs, fixed remodeling, and functional remodeling. Area under the curve (AUC) was determined. Sensitivity and specificity were also determined. A *P* value of <.05 was deemed significant.

## Results

A total of 40 patients were included (average age 59.2±12.0 years; 29 male [72.5%]). The average AF duration was 22.7±12.1 months with an LA size of 44.5±6.7 mm. Baseline characteristics are presented in Online Supplemental Table 1. All procedures were performed under local anesthesia and sedation. The average procedure duration was 149.0±28.8 minutes, with an average fluoroscopy and dose-area product of 3.5±1.3 minutes and 48.6±22.9 cGy·cm^2^, respectively. The average mapping time was 31.2±9.5 minutes. All patients underwent direct current cardioversion to SR and had BV maps created in SR. Of the 40 patients, 9 (22.5%) had AF recurrence post-cardioversion during mapping and required further cardioversion to restore SR and allow completion of mapping in SR. All patients underwent PVI with wide area circumferential ablation procedures. No further ablation was performed. No complications were encountered in this cohort.

### Scar assessment

In the 40 patients, 200 voltage maps were reviewed (40 AF OV, 40 SR BV 600 ms CS pacing, 40 SR BV 250 ms CS pacing, 40 SR BV 600 ms LAA pacing, and 40 SR BV 250 ms LAA pacing). There was no significant difference in the voltage measurements obtained when comparing coregistered points between SR BV 600 ms CS pacing maps and SR BV 600 ms LAA pacing maps (0.03±0.03 mV; *P*=.42). This was also true for SR BV 250 ms CS pacing maps and SR BV 250 ms LAA pacing maps (0.03±0.02 mV; *P*=.45). Thereby, SR BV 600 ms and SR BV 250 ms CS pacing maps were used for the analysis.

Across a total of 790,764 points with an average of 6589.7±315.5 points per map, there was a significant difference in average voltage between the 3 voltage maps. The average voltage was lower on AF OV maps than on SR BV 600 ms (0.49±0.76 mV AF OV vs 0.72±0.65 mV SR BV 600 ms; *P*<.0001) and SR BV 250 ms (0.49±0.76 mV AF OV vs 0.52±0.84 mV SR BV 250 ms; *P*=.10) maps. The average voltage was also lower on SR BV 250 ms maps than on SR BV 600 ms maps (0.52±0.84 mV SR BV 250 ms vs 0.72±0.65 mV SR BV 600 ms; *P*<.0001).

The proportion of the LA area occupied by LVZs was significantly different between the 3 voltage maps (30.4%±8.2% AF OV, 20.3%±7.4% SR BV 600 ms, and 29.4%±6.7% SR BV 250 ms; *P*=.001). The proportion of the LA area occupied by LVZs was greater on SR BV 250 ms maps than on SR BV 600 ms maps (29.4%±6.7% SR BV 250 ms vs 20.3%±7.4% SR BV 600 ms; *P*=.001). Comparing coregistered points between AF OV and SR BV 600 ms maps, an average difference in voltage of 0.21±0.09 mV was observed (*P*<.001). Comparing coregistered points between AF OV and SR BV 250 ms maps, an average difference in voltage of 0.06±0.04 mV was observed (*P*=.10).

A majority of LVZs (89.2%) identified on AF OV maps that did not correlate with LVZs on SR BV 600 ms maps correlated with LVZs on SR BV 250 ms maps. These LVZs thereby represented sites of functional remodeling. The remaining LVZs (10.8%) identified on AF OV maps that did not correlate with LVZs on SR BV 600 ms or LVZs on SR BV 250 ms maps, that is, AF-only LVZs.

LVZs identified on all 3 maps, which were defined as fixed remodeling, were more commonly mapped to the anterior (34.3%), posterior (31.2%), and lateral (25.4%) walls. Anatomically, functional remodeling sites more frequently involved the anterior (39.7%) and posterior (28.2%) walls and the anteroseptum (21.3%).

### Relationship between PF and voltage

PF values for each region and their differences are presented in Table 1 and Online Supplemental Figures 2A and 2B. A significant difference in PF was noted between sites of nLVZs, fixed remodeling, and functional remodeling (278.5±58.6 Hz nLVZs, 174.8±42.6 Hz fixed remodeling, and 230.6±41.2 Hz functional remodeling; *P*<.001). PF at fixed remodeling sites was significantly different from PF at functional remodeling sites (174.8±42.6 Hz fixed remodeling vs 230.6±41.2 Hz functional remodeling; *P*<.001), nLVZs (174.8±42.6 Hz fixed remodeling vs 278.5±58.6 Hz nLVZs; *P*<.001), and AF-only LVZs (174.8±42.6 Hz fixed remodeling vs 278.4±64.2 Hz AF-only LVZs; *P*<.001). PF at functional remodeling sites was also significantly different from PF at nLVZs (230.6±41.2 Hz functional remodeling vs 278.5±58.6 Hz nLVZs; *P*<.001) and AF-only LVZs (230.6±41.2 Hz functional remodeling vs 278.4±64.2 Hz AF-only LVZs; *P*<.001). There was no significant difference in PF at nLVZs and AF-only LVZs (278.5±58.6 Hz nLVZs vs 278.4±64.2 Hz AF-only LVZs; *P*=.89).

**Table 1:** Comparison of peak frequency in accordance with the underlying voltage zone

Voltage zone	Comparison region	*P*
Fixed remodeling	nLVZs	<.001
174.8±42.6 Hz	278.5±58.6 Hz	
Fixed remodeling	AF-only LVZs	<.001
174.8±42.6 Hz	278.4±64.2 Hz	
Fixed remodeling	Functional remodeling	<.001
174.8±42.6 Hz	230.6±41.2 Hz	
Functional remodeling	nLVZs	<.001
230.6±41.2 Hz	278.5±58.6 Hz	
Functional remodeling	AF-only LVZs	<.001
230.6±41.2 Hz	278.4±64.2 Hz	
Functional remodeling	Fixed remodeling	<.001
230.6±41.2 Hz	174.8±42.6 Hz	
AF-only LVZs	nLVZs	.89
278.4±64.2 Hz	278.5±58.6 Hz	

AF = atrial fibrillation; LVZ = low voltage zone; nLVZ = non–low voltage zone.

### Diagnostic accuracy of PF in characterizing scar in AF

PF showed a high diagnostic accuracy in predicting fixed remodeling with an AUC of 0.90 (*P*<.001). The optimal PF cutoff was ≤214 Hz for fixed remodeling with a sensitivity of 81.7% (95% confidence interval [CI] 70.2%–91.9%) and a specificity of 80.0% (95% CI 71.2%–90.2%). With regard to functional remodeling, PF showed an AUC of 0.76 (*P*<.001) with an optimal cutoff of ≤236 Hz with a sensitivity of 71.6% (95% CI 66.1%–73.0%) and a specificity of 65.8% (95% CI 59.1%–67.5%) and PF ≥215 Hz with a sensitivity of 70.1% (95% CI 68.3%–73.1%) and a specificity of 64.4% (95% CI 62.1%–66.2%). PF showed a high diagnostic accuracy in predicting nLVZs with an AUC of 0.71 (*P*<.001) with an optimal cutoff of ≥244 Hz with a sensitivity of 71.7% (95% CI 68.1%–75.3%) and a specificity of 60.8% (95% CI 58.2%–62.3%). PF also showed a high diagnostic accuracy in predicting AF-only LVZs with an AUC of 0.71 (*P*<.001) with an optimal cutoff of ≥242 Hz with a sensitivity of 71.3% (95% CI 68.1%–75.3%) and a specificity of 60.8% (95% CI 58.2%–62.3%). These findings are summarized in Table 2. The sensitivity (<60.0%) and specificity (<50.0%) of predicting fixed remodeling become lower once the PF is <187 Hz. Thereby, a PF of ≥187 Hz can be used as the lower cutoff for fixed remodeling. PF measurements <187 Hz can be used to exclude sites of potential far-field signals within LVZs rather than fixed remodeling.

**Table 2:** Diagnostic accuracy of peak frequency in differentiating the underlying substrate into nLVZs, fixed remodeling, and functional remodeling

Variable	nLVZs	AF-only LVZs	Fixed remodeling	Functional remodeling
PF (Hz), mean ± SD	278.5±58.6	278.4±64.2	174.8±42.6	230.6±41.2
AUC	0.71	0.71	0.90	0.76
Optimal PF cutoff (Hz)	≥244	≥242	≤214	≤236
Sensitivity (95% CI) (%)	71.7 (68.1–75.3)	71.3 (68.1–75.3)	81.7 (70.2–91.9)	71.6 (66.1–73.0)
Specificity (95% CI) (%)	60.8 (58.2–62.3)	60.8 (58.2–62.3)	80.0 (71.2–90.2)	65.8 (59.1–67.5)

AF = atrial fibrillation; AUC = area under the curve; CI = confidence interval; LVZ = low voltage zone; nLVZ = non–low voltage zone; PF = peak frequency.

### PF as a predictor of underlying remodeling in accordance with voltage

**Figure 3:**
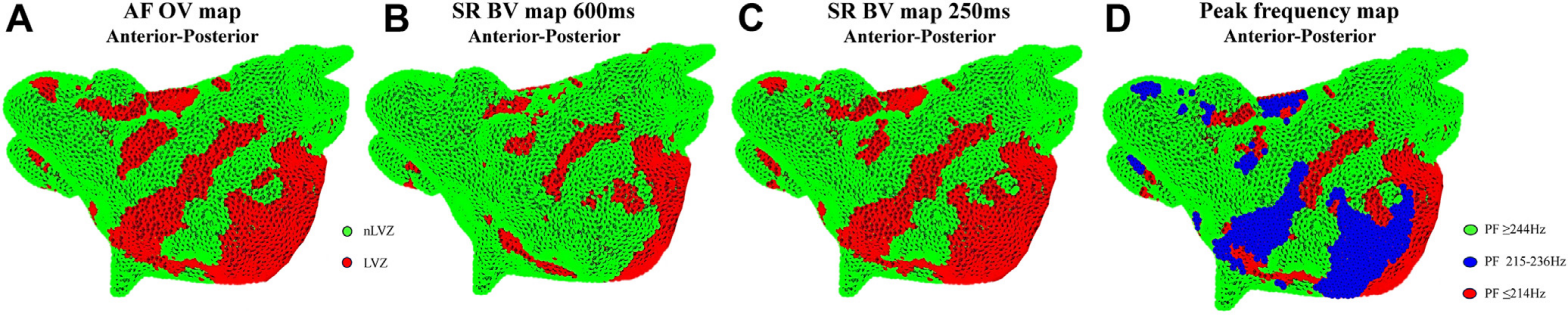
Voltage maps and peak frequency (PF) maps re-created in MatLab. **A:** Omnipolar voltage (OV) map created in atrial fibrillation (AF) in an anterior-posterior view. **B**: Bipolar voltage (BV) map created in sinus rhythm (SR) with atrial pacing at a pacing interval (PI) of 600 ms in an anterior-posterior view. **C:** BV map created in SR with atrial pacing at a PI of 250 ms in an anterior-posterior view. Low voltage zones (LVZs) are shown in *red*, and non–low voltage zones (nLVZs) are shown in *green*. **D:** PF map in an anterior-posterior view, created from PF measurements obtained in AF. When comparing the maps, the OV map in AF resembles the BV map in SR at a PI of 250 ms and both these maps demonstrate additional LVZs not seen on the BV map in SR at a PI of 600 ms, particularly anterior to the right lower pulmonary vein and anterior to the left atrial appendage. These additional LVZs represented sites of functional remodeling. LVZs seen across all the 3 maps represent sites of fixed remodeling. The PF map can effectively identify sites of fixed remodeling (PF ≤214 Hz represented in *red*) and functional remodeling (PF 215–236 Hz represented in *blue*). The OV map in AF demonstrated additional LVZs that were not seen on either of the SR maps. These sites demonstrated a PF of ≥244 Hz (represented in *green*) that was the same PF as obtained at nLVZs.

**Figure 4:**
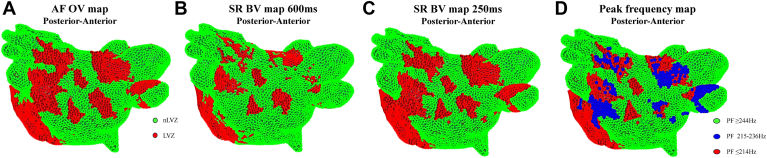
Voltage maps and peak frequency (PF) maps re-created in MatLab. **A:** Omnipolar voltage (OV) map created in atrial fibrillation (AF) in a posterior-anterior view. **B:** Bipolar voltage (BV) map created in sinus rhythm (SR) with atrial pacing at a pacing interval (PI) of 600 ms in a posterior-anterior view. **C:** BV map created in SR with atrial pacing at a PI of 250 ms in a posterior-anterior view. Low voltage zones (LVZs) are shown in *red*, and non–low voltage zones (nLVZs) are shown in *green*. **D:** PF map in a posterior-anterior view, created from PF measurements obtained in AF. When comparing the maps, the OV map in AF resembles the BV map in SR at a PI of 250 ms and both these maps demonstrate additional LVZs not seen on the BV map in SR at a PI of 600 ms, particularly posterior to the left lower pulmonary vein and posterior to the right upper pulmonary veins. These additional LVZs thereby represented sites of functional remodeling. LVZs seen across all the 3 maps represented sites of fixed remodeling. The PF map can effectively identify sites of fixed remodeling (PF ≤214 Hz represented in *red*) and functional remodeling (PF 215–236 Hz represented in *blue*). The OV map in AF demonstrated additional LVZs that were not seen on either of the SR maps. These sites demonstrated a PF of ≥244 Hz (represented in *green*) that was the same PF as obtained at nLVZs.

**Figure 5:**
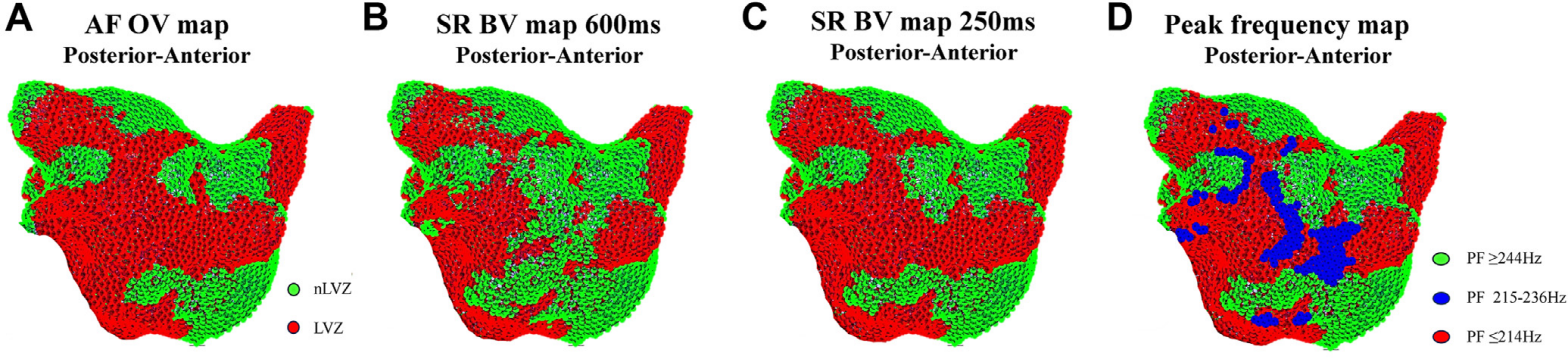
Voltage maps and peak frequency (PF) maps re-created in MatLab. **A:** Omnipolar voltage (OV) map created in atrial fibrillation (AF) in a posterior-anterior view. **B:** Bipolar voltage (BV) map created in sinus rhythm (SR) with atrial pacing at a pacing interval (PI) of 600 ms in a posterior-anterior view. **C:** BV map created in SR with atrial pacing at a PI of 250 ms in a posterior-anterior view. Low voltage zones (LVZs) are shown in *red*, and non–low voltage zones (nLVZs) are shown in *green*. **D:** PF map in a posterior-anterior view, created from PF measurements obtained in AF. When comparing the maps, the OV map in AF resembles the BV map in SR at a PI of 250 ms and both these maps demonstrate additional LVZs not seen on the BV map in SR at a PI of 600 ms, particularly the mid posterior wall. These additional LVZs thereby represented sites of functional remodeling. LVZs seen across all the 3 maps represented sites of fixed remodeling. The PF map can effectively identify sites of fixed remodeling (PF ≤214 Hz represented in *red*) and functional remodeling (PF 215–236 Hz represented in *blue*). The OV map in AF demonstrated additional LVZs that were not seen on either of the SR maps. These sites demonstrated a PF of ≥244 Hz (represented in *green*) that was the same PF as obtained at nLVZs.

Using the optimal cutoff of PF for fixed remodeling sites, a frequency of ≤214 Hz was shown to be a strong predictor of fixed remodeling (odds ratio 17.7; 95% CI 16.8–18.6; *P*<.001). For fixed remodeling, the model’s fit was significantly improved with the inclusion of a PF of ≤214 Hz as a predictor (χ^2^ = 17594.39; *P*<.001). For nLVZs, a PF of ≥244 Hz was shown to be a strong predictor of nLVZs (odds ratio 3.9; 95% CI 3.8–4.1; *P*<.001). For nLVZs, the model’s fit was significantly improved with the inclusion of a PF of ≥244 Hz as a predictor (χ^2^ = 5405.2; *P*<.001). For functional remodeling, a PF between 215 and 236 Hz was shown to be a strong predictor of functional remodeling (odds ratio 2.8; 95% CI 2.7–3.0; *P*<.001). For functional remodeling, the model’s fit was significantly improved with the inclusion of a PF of 215–236 Hz as a predictor (χ^2^ = 2216.4; *P*<.001). These findings are summarized in Table 3, Figures 3A–3D, Figures 4A–4D, and Figures 5A–5D.

**Table 3:** Results of binary logistic regression

Heart region	Peak frequency (Hz)	B	Standard error	Wald	*P*	Odds ratio	95% CI for odds ratio
nLVZ	≥244	1.36	0.019	4916.11	<.001	3.91	3.77–4.07
Fixed remodeling	≤214	2.87	0.025	13091.35	<.001	17.67	16.82–18.56
Functional remodeling	215–236	1.04	0.022	2277.25	<.001	2.83	2.71–2.95

CI = confidence interval; nLVZ = non–low voltage zone.

Comparing the PF findings between patients on amiodarone and those who were not on amiodarone, no difference in PF findings was found.

### Follow-up

All patients completed 12 months of follow-up with Holter monitoring at 6 and 12 months. Of the 40 patients, 25 (62.5%) were free of AF/AT during follow-up.

## Discussion

This study sought to investigate whether a novel parameter, PF obtained in AF, could aid in better characterization of the substrate in AF by delineating LVZs into fixed and functional remodeling sites alongside identifying sites of potential underestimation of voltage due to mapping in AF. The main findings of the study include the following:

• While OV mapping helps mitigate the effects of wavefront collision and EGM fractionation,^12^^,^^13^ both average voltages and voltages at coregistered points recorded in AF OV maps measured lower than those observed in SR at PIs of 600 and 250 ms. However, these differences were not significant when comparing to SR at a PI of 250 ms, suggesting that AF OV maps correlate better with SR maps obtained at higher atrial rates and thereby considering both fixed and functional remodeling.
• PF mapping effectively differentiated between nLVZs, fixed remodeling, and functional remodeling sites. PFs of ≤214 and ≥244 Hz were highly predictive of fixed remodeling and nLVZs, respectively. A PF between 215 and 236 Hz was highly predictive of functional remodeling. Thereby, the combination of OV and PF measurements in AF will more accurately allow the characterization of the substrate in AF.
• AF OV maps identified additional LVZs that were not seen on SR BV 600 ms and SR BV 250 ms maps, which is possible because of the underestimation of voltage due to mapping in AF. This is most likely the case as the PF values at these sites were not significantly different from the PF values obtained at nLVZs in AF OV maps. Thereby, PF can also be used to identify sites with potentially overestimated LVZs in AF.

OV mapping was developed as a tool to more accurately identify LVZs, minimizing distortions from wavefront collisions and bypassing the necessity for a perpendicular wavefront of activation relative to the dipoles.^12^ This technique yields voltages that more faithfully mirror those observed during SR pacing.^13^ Notably our research reveals that despite the enhanced clarity offered by OV mapping, both average and site-matched voltages are lower than those observed during SR pacing. Furthermore, the proportion of the LA exhibiting LVZs in AF OV maps is more extensive than that observed during SR pacing. In the AF OV maps, average voltages were the lowest. While SR BV 250 ms maps exhibited higher voltages than did AF OV maps, the difference was not significant. The voltage in SR BV 250 ms maps was still lower than that in SR BV 600 ms maps. This stepwise voltage decrease, more pronounced with increased pacing rates, suggests that low voltages are not solely attributed to fixed remodeling. Instead, certain susceptible regions may undergo rate-dependent proarrhythmic electrophysiological changes at faster pacing rates. We have designated these regions as functional remodeling sites. Our data also highlight that an exclusive reliance on SR voltage mapping could overlook functional remodeling, potentially omitting a key therapeutic target resulting in inferior therapeutic responses. Our group has shown that sites of functional remodeling colocate to pivot points and enhanced conduction velocity (CV) heterogeneity sites that are precursors for reentry formation.^19^ We have also shown that calibrating computational models to a PI of 250 ms and thereby considering functional remodeling results in an increase in rotational activity burden in AF.^19^ These findings support that functional remodeling plays a potential mechanistic role in AF. This study has shown that PF mapping provides an additional approach to further evaluate the mechanistic importance of functional remodeling in AF and thereby allow the potential identification of novel ablation targets.

Several ablation strategies have been explored in AF. Recent trials have proposed a role for substrate modification, whereby atrial scar is homogenized as an ablation strategy in AF.^8^, ^14^^,^^20^ The ERASE-AF trial^8^,^8^ demonstrated an improvement in the success rate of persistent AF ablation when comparing PVI plus substrate modification with PVI-only ablation. However, there are limitations associated with this trial that need to be recognized. First, as randomization occurred before the voltage map, the substrate modification group included patients who had no underlying substrate and thereby underwent only PVI. Thereby, the true effect of a substrate modification approach in addition to PVI is only elicited in subgroup analysis. Second, when comparing the success rate of PVI in the substrate modification group with the success rate of PVI in the control group, the success rate of PVI in the substrate modification group was greater. It is unclear why this is; however, it has been argued that as the operators were not blinded to the group allocation at the time of PVI, this resulted in a biased approach to PVI that accounts for the discrepancy in PVI outcomes. Regardless of these limitations, on the basis of the subgroup analysis, there is an indication that substrate modification of LVZs does positively affect the success rate of persistent AF ablation. While most studies use voltage maps to assess LVZs, other trials have evaluated the role of cardiac magnetic resonance imaging (MRI) to guide fibrosis ablation. The DECAAF II randomized clinical trial,^21^ which compared PVI plus cardiac MRI–guided fibrosis ablation with PVI-only ablation, did not result in lower arrhythmia recurrence. These findings were also demonstrated in the ALICIA trial.^22^ Whether this indicates that fibrosis ablation is not an effective ablation strategy for persistent AF or that the use of cardiac MRI for the identification of fibrosis is not an effective approach remains unclear. Regardless, based on the subgroup analysis from the ERASE-AF trial, substrate modification with PVI improved the success rate of AF ablation, with lower rates of arrhythmia recurrence in patients with underlying LVZs compared to PVI-only ablation. These trials have focused on targeting sites of fixed remodeling and not functional remodeling. With the potential role of functional remodeling in AF, being able to identify these sites provides an additional target for ablation. The benefit of substrate modification of fixed and functional remodeling with ablation does need to be further evaluated in randomized controlled trials.

Multiple mechanisms may contribute to the formation of functional remodeling sites. Rapid pacing can lead to progressive, staggered failure of recovery of Na_v_1.5 channels, reducing peak I_Na_.^23^ Such changes alone could reduce voltage in addition to inducing slower conduction, which itself has been linked to the formation of LVZs.^24^^,^^25^ Moreover, swift atrial pacing can trigger spatial action potential duration alternans that can precipitate conduction block^26^ and possible subsequent LVZs. Certain regions might exhibit minimal fibrosis, characterized by a modest integration with fibroblasts, resulting in a normal voltage at modest activation rates. However, this subtle sink enhancement, when combined with a decrease in source secondary to I_Na_ reduction due to rapid atrial pacing, could potentially contribute to marked reductions in CV, subsequent block, and LVZs.

It should be noted that functional remodeling likely only partially accounts for the observed voltage discrepancies. Even though not significant, AF OV maps show lower voltages than do SR BV 250 ms maps; it is unclear whether this reflects overestimated LVZs or truly denotes more pronounced low voltage sites during AF than during rapid atrial pacing. Such disparities in AF could stem from its erratic activation patterns shaped by reentrant circuits, multiple wavelets, and potential rotors, leading to increased functional block and reduced CV.^27^

Mapping in SR to assess fixed and functional remodeling is the gold standard. However, mapping in SR can be time-consuming and adds additional time to the procedure. Further to this, it is dependent on restoring SR, which is not always possible or when possible, pacing at higher atrial rates can induce AF, resulting in the need for further cardioversion. In this study, a proportion of patients needed >1 cardioversion and there was an additional mapping time of ∼30 minutes. Being able to identify fixed and functional remodeling in AF would result in not requiring additional mapping in SR and as a result overcome the above. In this study, it has been shown that the use of PF in AF allows better characterization of scar in AF and potentially avoiding the need for mapping in SR.

### Insight from PF analysis

LVZs play a pivotal role in AF, and their ablation has offered promising results in the challenging domain of AF treatment.^8^ While cardiac MRI has often associated these LVZs with fibrotic regions,^6^ discerning more information about the underlying substrate from voltage readings alone is novel.

Our investigation underscores the potential of PF analysis to elucidate substrate differences within LVZs, allowing us to delineate between fixed and functional remodeling territories. This offers a richer understanding of LVZ composition, obviating the need for supplementary imaging or labor-intensive mapping at various rapid PIs. Further to this, PF has the potential to exclude sites of overestimated LVZs in AF, thereby limiting ablation to sites of true LVZs and better guide prognostic assessment with regard to freedom from AF/AT based on scar burden.^28^

At its core, PF analysis leverages wavelet analysis, distinguishing it from DF analysis, which uses a fast Fourier transform approach. The latter decomposes an intracardiac EGM into constituent sine and cosine waves, prioritizing amplitude but sacrificing temporal resolution and obscuring the spectrum of inherent frequencies. In contrast, PF analysis uses a foundational “mother wavelet,” which undergoes dynamic stretching and shifting to generate a series of “daughter wavelets.” Evaluating the congruence of these wavelets across time intervals reveals a complex spectrum of frequencies embedded within each EGM. This can be pictured as an orchestra; DF analysis identifies the loudest instrument playing whereas wavelet analysis distinguishes and pinpoints each individual instrument. As this study indicates, analyzing PF can shed new light into substrate characteristics, a perspective previously unexplored.

Our insights intimate that fixed remodeling sites exhibit a subdued PF relative to other sites. This observation may stem from multiple etiologies: scarred terrains might be bereft of cardiac conduction, or they might necessitate circuitous conduction paths, yielding protracted EGMs characterized by more gradual voltage transitions and thus lower frequencies. In stark contrast, healthy tissues facilitate swift conduction, producing EGMs marked by pronounced voltage fluctuations and higher PFs. Functional remodeling sites emerge as intermediate landscapes, where conduction slowing, functional blocks, and ion channel modulations may induce discernible alterations in bipolar EGMs, though not as profound as those manifests in scarred regions. Furthermore, we identified the potential of PF analysis in pinpointing sites where LVZs might have been overestimated, termed as AF-only LVZs. Such territories, although seemingly compromised by low voltages, radiate high PFs, hinting at healthy underlying tissue.

Our exploration marks an initial step in understanding functional remodeling zones. Further research could illuminate their role in sustaining AF and the ramifications of their ablation. A more detailed understanding of these regions may pave the way for refined targeting, potentially via radiofrequency ablation or even pharmacological avenues.

### Limitations

We used 2 PIs to create SR voltage maps. While this offers a broad insight and is likely to capture restitution effects, it is conceivable that voltage characteristics may behave differently outside these pacing rates. This requires further evaluation.

Our cohort size is modest, although on par with other mechanistic studies. Further to this, even though the patient number is modest, the data analysis consisted of >700,000 points, which should be sufficient for evaluating PF in characterizing the substrate in AF.

We have yet to assess the impact of modifying ablation strategies on the basis of our findings. Randomized controlled trials are required to evaluate whether targeting fixed and functional remodeling would result in an improvement in AF ablation success rate. Randomized controlled trials are also required to evaluate whether the use of PF to distinguish these sites and then targeting these using the PF maps results in an improvement in AF ablation success rates.

## Conclusion

In the challenging domain of AF management, gaining a detailed understanding of the substrate targeted for modification is vital. This study presents the innovative benefits of combining OV mapping with a novel parameter PF when evaluating the substrate in AF. The use of PF enables identification and differentiation between fixed remodeling sites, functional remodeling sites, and sites with suspected overestimated low voltages in AF. While our results highlight the potential of these techniques in substrate characterization, they also shed light on the intricacies of interpreting LVZs, notably the risk of overlooking functional remodeling sites if relying solely on SR voltage mapping. A combination of mechanistic research and clinical trials is essential to further deepen our insights and achieve tangible clinical results.

## Figures and Tables

**Supplemental Figure 2:**