# Supplementary material for: Peak frequency can be effectively used to characterize scar in atrial fibrillation
Source: Heart Rhythm O2. 2025 Jan 9;6(4):434–43. doi: 10.1016/j.hroo.2024.12.011 (PMC12047463; doi:10.1016/j.hroo.2024.12.011)
Supplement: Supplementary Data [file mmc1.docx]

**Supplemental Figure Legend**

***Supplemental Figure 1-*** Schematic demonstrating wavelet analysis.

***Supplemental Figure 2A-B-*** This graph shows mean peak frequency plotted against binned voltage amplitude taken at 0.01mV intervals. ***A)*** SR BV maps created at a PI of 600ms, which show that PF is lower in areas of LVZs whilst higher in areas of nLVZs. The dashed line represents the cut off for LVZs (<0.5mV). ***B)*** SR BV maps created at a PI of 250ms, also demonstrates that PF is lower in areas of LVZs whilst higher in areas of nLVZs. In addition, in areas of functional remodeling (highlighted by square box) i.e. LVZs on SR BV 250ms maps but nLVZs on SR BV 600ms maps have a higher PF compared to LVZs but lower than nLVZs.
