# Supplementary material for: Peak frequency can be effectively used to characterize scar in atrial fibrillation
Source: Heart Rhythm O2. 2025 Jan 9;6(4):434–43. doi: 10.1016/j.hroo.2024.12.011 (PMC12047463; doi:10.1016/j.hroo.2024.12.011)
Supplement: Supplemental Table 1 [file mmc2.docx]

| **Baseline characteristics** | **Cohort n=40** |
| --- | --- |
| Age yrs. mean ± SD | 59.2±12.0 |
| Male n (%) | 29 (72.5) |
| Diabetes mellitus n (%) | 7 (17.5) |
| Hypertension n (%) | 7 (17.5) |
| TIA/CVA^Ƭ^ n (%) | 1 (2.5) |
| Ischaemic heart disease n (%) | 6 (15.0) |
| Cardiac surgery n (%) | 0 (0) |
| Cardiomyopathy n (%) | 16 (40.0) |
| BMI^Ŧ^ kg/m^2^ n (%)  20-30  31-40  >40 | 18 (45.0)  20 (50.0)  2 (5.0) |
| Obstructive sleep apnoea n (%) | 6 (15.0) |
| Left ventricular EF^δ^ ≥ 55% n (%) | 22 (55.0) |
| LA size mm n (%)  30-40  41-50  >50 | 18 (45.0)  17 (42.5)  5 (12.5) |
| AF duration months, mean ± SD | 22.7±12.1 |
| Previous AT ablation n (%)  Cavo-tricuspid isthmus-dependent flutter | 3 (7.5) |
| Current anti-arrhythmic or rate-controlling strategy n (%)  Beta-blockers including Sotalol  Amiodarone  Flecainide  Calcium channel blocker  Digoxin | 32 (80.0)  11 (27.5)  0 (0)  1 (2.5)  4 (10.0) |
| Current anticoagulation strategy n (%)  Warfarin  Direct oral anticoagulants  ^Ƭ^TIA/CVA= Transient ischemic attack/Cerebrovascular accident  ^Ŧ^BMI- Body mass index  ^δ^EF- Ejection fraction | 0 (0)  40 (100) |

***Supplemental Table 1-*** *Baseline Characteristics*
